# Successful lung transplantation in an HIV seropositive patient with desquamative interstitial pneumonia: a case report

**DOI:** 10.1186/s12890-018-0727-0

**Published:** 2018-10-16

**Authors:** Shaun Ong, Robert D Levy, John Yee, Nilu Partovi, Andrew Churg, Philippe Roméo, Jean Chalaoui, Roland Nador, Alissa Wright, Hélène Manganas, Christopher J Ryerson

**Affiliations:** 1 Department of Medicine, University of British Columbia, 2775 Laurel Street, 10th Floor, Vancouver, BC V5Z 1M9 Canada; 2 Lung Transplant Program, Vancouver General Hospital, 2775 Laurel Street, 5th Floor, Vancouver, BC V5Z 1M9 Canada; 3 Division of Respirology, Department of Medicine, University of British Columbia, 2775 Laurel Street, 7th Floor, Vancouver, BC V5Z 1M9 Canada; 4 Division of Thoracic Surgery, Department of Surgery, University of British Columbia, 950 West 10th Avenue, Vancouver, BC V5Z 1M9 Canada; 5 Department of Pharmacology, University of British Columbia, 217 – 2176 Health Sciences Mall, Vancouver, BC V6T 1Z3 Canada; 6 Vancouver Coastal Health Research Institute, University of British Columbia, 2635 Laurel Street, Vancouver, BC V5Z 1M9 Canada; 7 Department of Pathology, University of British Columbia, Room G227 – 2211 Wesbrook Mall, Vancouver, BC V6T 2B5 Canada; 8 Centre for Heart Lung Innovation, St. Paul’s Hospital, Room 166 – 1081 Burrard Street, Ward 8B, Vancouver, BC V6Z 1Y6 Canada; 9 Department of Pathology, Centre Hospitalier de l’Université de Montréal, University of Montreal, 1051 Sanguinet Street, Montreal, QC H2X 3E4 Canada; 10 Department of Radiology, Centre Hospitalier de l’Université de Montréal, University of Montreal, 1051 Sanguinet Street, Montreal, QC H2X 3E4 Canada; 11 Division of Infectious Diseases, Department of Medicine, University of British Columbia, 452D – 2733 Heather Street, Vancouver, BC V5Z 3J5 Canada; 12 Division of Respirology, Department of Medicine, Centre Hospitalier de l’Université de Montréal, 1051 Sanguinet Street, Montreal, QC H2X 3E4 Canada

**Keywords:** HIV, Lung transplant, Desquamative interstitial pneumonia, Immunosuppression

## Abstract

**Background:**

Until recently, lung transplantation was not considered in patients with human immunodeficiency virus (HIV). HIV seropositive patients with suppressed viral loads can now expect long-term survival with the advent of highly active antiretroviral therapies (HAART); however, HIV remains a relative contraindication to lung transplantation. We describe, to our knowledge, the first HIV seropositive lung transplant recipient in Canada. We also review the literature of previously reported cases of solid-organ transplantation in patients with HIV with a focus on immunosuppression considerations.

**Case presentation:**

A 48-year old man received a bilateral lung transplant for a diagnosis of desquamative interstitial pneumonia (DIP) attributed to cigarette and cannabis smoking. His control of HIV infection pre-transplant was excellent on HAART, and he had no other contraindications to lung transplantation. The patient underwent bilateral lung transplantation using basiliximab, methylprednisolone, and mycophenolate mofetil (MMF) as induction immunosuppression. He was maintained on MMF, prednisone, and tacrolimus thereafter, and restarted his HAART regimen immediately post-operatively. His post-transplant course was complicated by Grade A1 minimal acute cellular rejection, as well as an enterovirus/rhinovirus graft infection. Despite these complications, his functional status and control of HIV infection remain excellent 24 months post-transplant.

**Conclusions:**

Our patient is one of only several HIV seropositive lung transplant recipients reported globally. With growing acceptance of transplantation in this population, there is a need for clarification of prognosis post-transplantation, as well as optimal immunosuppression regimens for these patients. This case report adds to the recent literature that suggests HIV seropositivity should not be considered a contraindication to lung transplantation, and that post-transplant patients with HIV can be managed safely with basiliximab, tacrolimus, MMF and prednisone.

## Background

Until recently, lung transplantation was not considered in patients with human immunodeficiency virus (HIV) given the poor prognosis associated with being HIV seropositive and the need for additional post-transplant immunosuppression. With the advent of highly active antiretroviral therapies (HAART) for HIV, seropositive patients with suppressed viral loads can now expect long-term survival. Despite these advances, HIV seropositivity remains a relative contraindication to lung transplantation per International Society for Heart and Lung Transplantation (ISHLT) guidelines [1], and there are few reports of HIV seropositive patients who have undergone lung transplantation [2–4].

Here, we describe, to our knowledge, the first HIV seropositive lung transplant recipient in Canada. We also provide a literature review of previously reported cases of solid-organ transplantation in patients with HIV, with a focus on immunosuppression considerations and prognosis.

## Case presentation

**Fig. 1:**
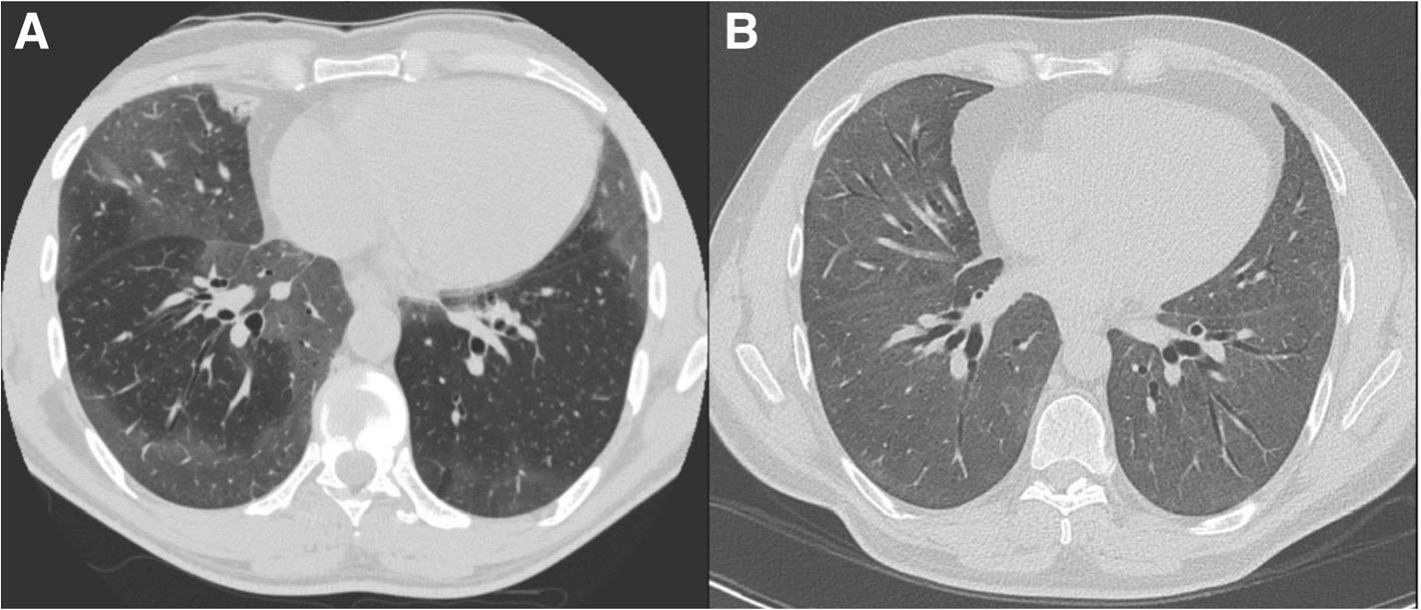
High-resolution computed tomography (HRCT) of the chest in axial cut from time of initial presentation **(a)**, and 2 years post-diagnosis **(b)**. Both HRCTs demonstrate multifocal ground glass consolidation, primarily in the lower lung zones, progressive over an 8-year period

**Fig. 2:**
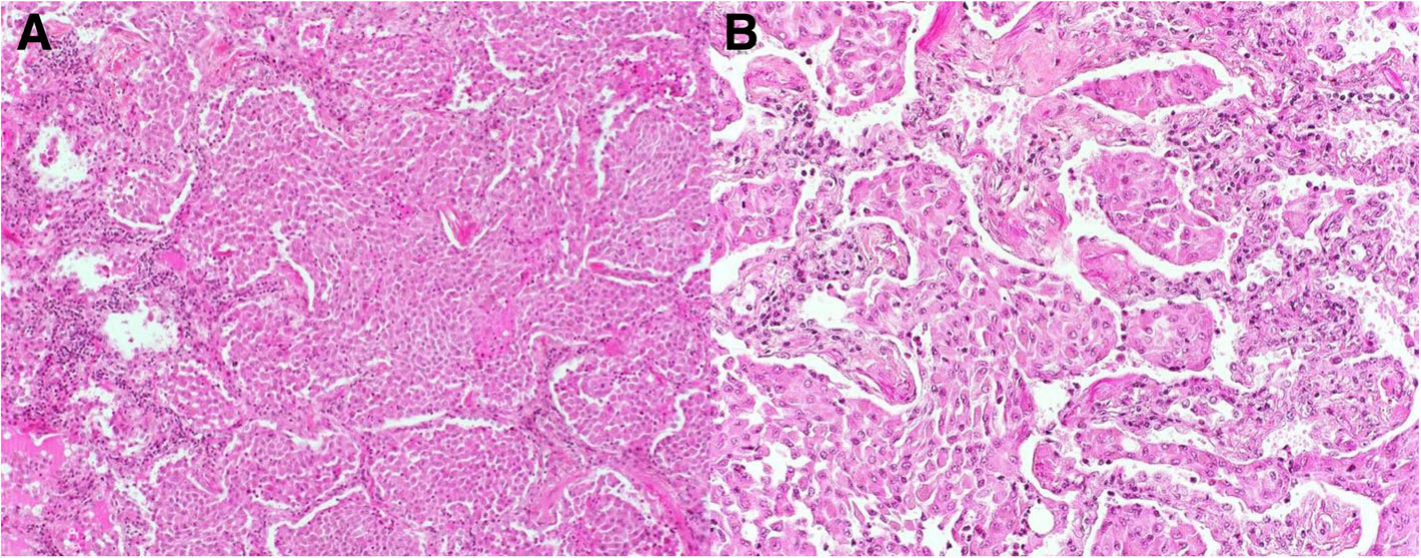
Haematoxylin phyloxin saffron stain at 100× **(a)**, and 200× **(b)** of surgical lung biopsy specimen showing filling of alveolar spaces by macrophages. There is mild interstitial inflammatory infiltrate

A 48-year-old man received a bilateral lung transplant for a diagnosis of desquamative interstitial pneumonia (DIP) that was attributed to smoking cigarettes and cannabis, both of which he ceased 20 months prior to transplantation. The diagnosis of DIP was based on compatible imaging and a prior surgical lung biopsy that was performed at the age of 44 years (Figs. 1 and 2).

The patient was diagnosed with HIV at the age of 38 years and had started HAART 20 months prior to transplantation at age 47. He achieved excellent control of his HIV infection, with a pre-transplant CD4^+^ cell count of 950 cells/μL, CD4^+^:CD8^+^ ratio of 0.69, no detectable viral load, and no history of AIDS-defining illnesses. His HIV was initially managed with cobicistat, elvitegravir, emtricitabine, and tenofovir, which were subsequently changed to abacavir, dolutegravir, and lamivudine when lung transplantation became a consideration due to pharmacokinetic interactions between cobicistat and tacrolimus [5–8].

The patient had progressively worsening dyspnea and lung function that prompted initiation of long-term prednisone and eventually mycophenolate mofetil (MMF), which were both prescribed in consultation with his HIV specialist. His interstitial lung disease continued to progress both clinically and radiographically (Fig. 1), with pre-transplant lung function showing a forced expiratory volume in 1 s (FEV_1_) 55% predicted, forced vital capacity (FVC) 50% predicted, and diffusing capacity for carbon monoxide (DLCO) 36% predicted. Prior to transplant, he required 2 l oxygen via nasal prongs at rest.

**Fig. 3:**
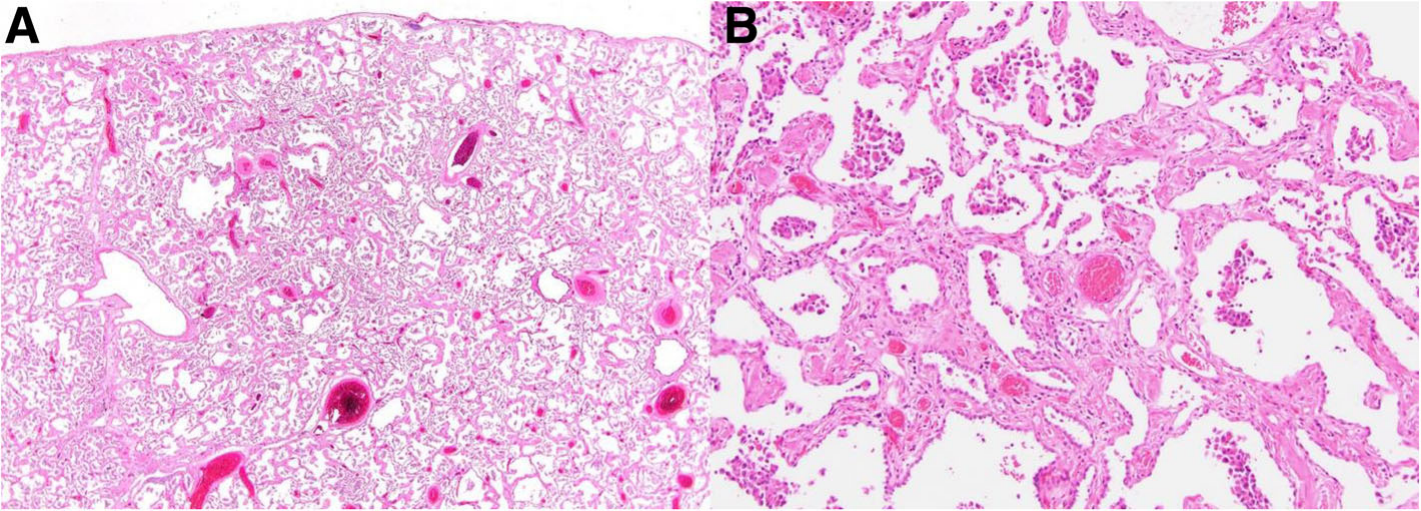
10× **(a)** and 100× **(b)** views of the explanted lungs. The number of airspace macrophages has considerably decreased compared to the surgical lung biopsy, and the process more closely resembles fibrotic non-specific interstitial pneumonia than desquamative interstitial pneumonia

His induction immunosuppression included basiliximab (20 mg on date of transplantation and post-operative day 4), methylprednisolone (500 mg at induction, 500 mg at reperfusion, and three doses of 125 mg on the day of transplant), and MMF (1 g pre-transplant). His explanted lungs still showed a pattern of DIP, but with a decreased number of airspace macrophages and a picture that in areas was more consistent with fibrotic nonspecific interstitial pneumonia (NSIP) (Fig. 3). A similar progression of DIP to an NSIP-like picture has been reported previously [9].

Post-transplant, his maintenance immunosuppression included MMF 1 g twice daily, prednisone 15 mg daily, and tacrolimus, dosed to a target trough level of 10-12 ng/mL. His previous HAART regimen was also continued. The patient had an uncomplicated course in hospital and was discharged from the intensive care unit on post-operative day 3 and discharged from hospital on day 18. He was maintained on daily valganciclovir 900 mg, trimethoprim-sulfamethoxazole 160-800 mg, and azithromycin 250 mg for prophylaxis against opportunistic infections. Valganciclovir was discontinued 6 months post-transplant as cytomegalovirus DNA remained undetectable in his serum. His tacrolimus trough levels were reduced to 8-10 ng/mL 10 months post-transplant. His prednisone dose was reduced to 10 mg and then subsequently 5 mg daily at 6- and 14-months post-transplant respectively.

He had an episode of Grade A1 minimal acute cellular rejection detected on surveillance transbronchial biopsy 3 months post-transplant. This was not treated, and there was no evidence of graft rejection on repeat biopsies 1 month later. He had an enterovirus/rhinovirus graft infection with significant allograft dysfunction 11 months post-transplant and received 3 daily doses of methylprednisolone 500 mg with a following prednisone dose of 60 mg daily, tapered by 5 mg every 5 days to a baseline dose of 10 mg thereafter. At the time of this infection, he also received a single dose of intravenous immunoglobulin 0.5 g/kg.

**Fig. 4:**
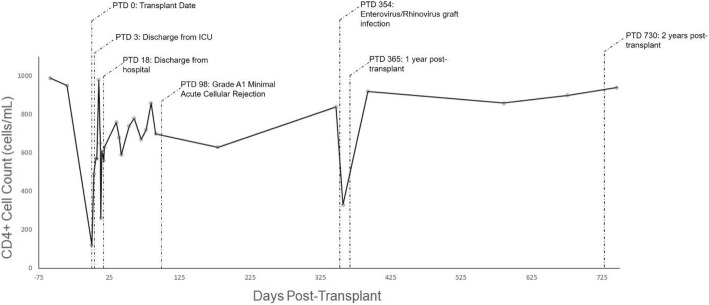
CD4^+^ cell count in relation to date of bilateral lung transplant. Significant events during the patient’s course are denoted by annotations with post-transplant day (PTD) specified

Post-transplant, the patient’s markers of HIV infection continue to be controlled, with CD4^+^ cell count nadir of 120 cells/μL on post-operative day 3. CD4^+^ count increased to 630 cells/μL at time of discharge from hospital, and was 940 cells/μL at 24 months post-transplant, with a CD4^+^:CD8^+^ ratio of 1.03, and undetectable viral load throughout (Fig. 4). He continues to have normal functional status, no dyspnea, normal lung function, and normal ambulatory oxygenation 24 months post-transplantation.

## Discussion and conclusions

There have been eight previously reported cases of lung transplantation in HIV seropositive patients globally [2–4]. Of the 5 patients who received transplantation more than 3 years ago, 4 have achieved 3-year survival, which is a comparable outcome to non-HIV seropositive patients [4, 10]. There are also numerous reports of HIV seropositive patients who have received heart transplants, the majority of which have had good outcomes since the introduction of HAART [11]. Observational data from renal and liver transplant populations have generally suggested that HIV seropositive patients have similar survival compared to seronegative patients if maintained on HAART [12–15]. Conversely, the largest cohort study of HIV seropositive liver and kidney recipients to date reported an increased risk of graft failure in recipients from both subpopulations [16], and a higher risk of mortality in liver recipients. Multiple other studies have also suggested a higher than expected risk of acute rejection in HIV seropositive kidney recipients [13, 15, 17, 18].

Until recently, induction immunosuppression was thought to increase the risks of adverse outcomes in HIV seropositive patients [19]. Modern immunosuppression regimens for induction and acute rejection in solid-organ transplant recipients rely on the inhibition of lymphocyte proliferation and activity, and occasionally the more widespread reduction of lymphocyte populations. This latter approach presents unique challenges in HIV seropositive transplant recipients given CD4^+^ cell populations and CD4^+^:CD8^+^ ratios are important prognostic factors in the management of HIV progression and treatment response [20–22]. This applies to both medications frequently used in the induction and maintenance phases of post-lung transplantation immunosuppression.

Induction therapies for lung transplant typically include anti-thymocyte globulin (ATG) and basiliximab. ATG is a polyclonal antibody that actively depletes T-lymphocytes. ATG reduces CD4^+^ cell counts immediately after treatment, and also causes prolonged suppression of CD4^+^:CD8^+^ ratios [23–25]. When used as induction immunosuppression in lung transplantation, ATG may increase overall survival by reducing graft loss despite also increasing the risk of infection [26]. In the largest cohort of HIV seropositive liver and kidney transplant recipients to date, ATG use in the first week post-kidney transplantation was associated with increased mortality and graft loss [13, 16]. Other studies in HIV seropositive renal transplant recipients have instead suggested that induction immunosuppression with ATG does not increase the risk of infection compared to HIV seronegative controls, and furthermore decreases the risk of graft loss and increases survival [19, 27]. Basiliximab is an IL-2 receptor antagonist that inhibits proliferation and differentiation of T-cells. IL-2 receptor antagonists continue to be the most frequently used induction agents in HIV seronegative lung transplant recipients [28]. Basiliximab use in induction for HIV seropositive renal transplant recipients has been associated with a lower incidence of delayed graft function, reduced graft loss, and a trend towards longer survival compared to no induction [19]. These findings therefore support the use of basiliximab in the induction phase for HIV seropositive lung and renal transplant recipients. Basiliximab may be a preferable induction agent for HIV seropositive patients given ATG’s adverse effects on CD4^+^ cell populations and its association with increased mortality and graft loss in HIV seropositive renal transplant populations. Our patient tolerated the use of basiliximab as induction therapy well, and had no major adverse events and long-term sequelae attributed to its use.

Maintenance immunosuppressive therapies post-lung transplantation in most centers typically include tacrolimus, MMF, and prednisone, which all exert cytostatic effects on T-cell populations via several mechanisms [29–35]. MMF also exerts apoptotic effects on CD4^+^ cells in vitro [36]. Despite these findings, neither HIV seropositive or seronegative patients treated with these medications exhibit sustained decreases in their absolute CD4^+^ cell counts or declines in their CD4^+^:CD8^+^ ratios [13, 18, 25, 36–40]. Cobicistat and the protease inhibitor medications increase plasma calcineurin inhibitor levels via cytochrome P450 3A and P-glycoprotein inhibition [5–8], necessitating close monitoring of drug levels when these medications are co-administered with tacrolimus. These interactions were avoided in our patient with alterations to his HAART. Based on this information, HIV seropositive lung transplant recipients can safely be managed with MMF and tacrolimus. Our patient received these medications, as well as prednisone, and had no long-term complications with respect to graft rejection or control of HIV over a 2-year period, which further suggests that HIV seropositive patients can tolerate this immunosuppressant regimen if their HAART is carefully tailored.

In summary, this is the first HIV seropositive lung transplant recipient in Canada to our knowledge, and one of only several patients reported globally. Our immunosuppression selection included basiliximab, corticosteroids, tacrolimus and MMF; a regimen that is supported by evidence drawn from kidney and liver transplant populations. This case report adds to the growing body of literature that these medications are safe in HIV seropositive patients undergoing lung transplantation, and that these patients can achieve long-term outcomes that are comparable to HIV seronegative patients. There is, however, a need for large-scale studies of this specific population given the lack of evidence in optimal immunosuppression for these patients, as well as their long-term prognosis. This is especially the case with future trends towards increasing acceptance of solid-organ transplantation in selected HIV seropositive patients.

## Abbreviations

ATG: Anti-thymocyte globulin
DIP: Desquamative interstitial pneumonia
DLCO: Diffusing capacity for carbon monoxide
FEV1: Forced expiratory volume in 1 s
FVC: Forced vital capacity
HAART: Highly active antiretroviral therapy
HIV: Human immunodeficiency virus
ISHLT: International Society for Heart and Lung Transplantation
MMF: Mycophenolate mofetil
NSIP: Nonspecific interstitial pneumonia
